# The role of collagen and crystallinity in the physicochemical properties of naturally derived bone grafts

**DOI:** 10.1093/rb/rbae093

**Published:** 2024-08-14

**Authors:** Øystein Øvrebø, Luca Orlando, Kristaps Rubenis, Luca Ciriello, Qianli Ma, Zoe Giorgi, Stefano Tognoni, Dagnija Loca, Tomaso Villa, Liebert P Nogueira, Filippo Rossi, Håvard J Haugen, Giuseppe Perale

**Affiliations:** Department of Chemistry, Materials and Chemical Engineering “Giulio Natta”, Politecnico di Milano, 20133 Milano, Italy; Department of Biomaterials, Institute of Clinical Dentistry, University of Oslo, 0318 Oslo, Norway; Material Biomimetic AS, 0349 Oslo, Norway; Industrie Biomediche Insubri SA, 6805 Mezzovico-Vira, Switzerland; Orlando Engineering & Consulting Srl, 20094 Corsico, Italy; Institute of Biomaterials and Bioengineering, Faculty of Natural Sciences and Technology, Riga Technical University, LV-1007 Riga, Latvia; Baltic Biomaterials Centre of Excellence, Headquarters at Riga Technical University, Riga, Latvia; Department of Chemistry, Materials and Chemical Engineering “Giulio Natta”, Politecnico di Milano, 20133 Milano, Italy; Department of Biomaterials, Institute of Clinical Dentistry, University of Oslo, 0318 Oslo, Norway; Department of Chemistry, Materials and Chemical Engineering “Giulio Natta”, Politecnico di Milano, 20133 Milano, Italy; Department of Chemistry, Materials and Chemical Engineering “Giulio Natta”, Politecnico di Milano, 20133 Milano, Italy; Institute of Biomaterials and Bioengineering, Faculty of Natural Sciences and Technology, Riga Technical University, LV-1007 Riga, Latvia; Baltic Biomaterials Centre of Excellence, Headquarters at Riga Technical University, Riga, Latvia; Department of Chemistry, Materials and Chemical Engineering “Giulio Natta”, Politecnico di Milano, 20133 Milano, Italy; Department of Biomaterials, Institute of Clinical Dentistry, University of Oslo, 0318 Oslo, Norway; Oral Research Laboratory, Institute of Clinical Dentistry, University of Oslo, 0318 Oslo, Norway; Department of Chemistry, Materials and Chemical Engineering “Giulio Natta”, Politecnico di Milano, 20133 Milano, Italy; Department of Biomaterials, Institute of Clinical Dentistry, University of Oslo, 0318 Oslo, Norway; Material Biomimetic AS, 0349 Oslo, Norway; Industrie Biomediche Insubri SA, 6805 Mezzovico-Vira, Switzerland; Faculty of Biomedical Sciences, University of Southern Switzerland, 6900 Lugano, Switzerland; Ludwig Boltzmann Institute for Experimental and Clinical Traumatology, 1200 Vienna, Austria

**Keywords:** xenograft, dental, orthopaedics, regenerative medicine, bone graft, collagen

## Abstract

Xenografts are commonly used for bone regeneration in dental and orthopaedic domains to repair bone voids and other defects. The first-generation xenografts were made through sintering, which deproteinizes them and alters their crystallinity, while later xenografts are produced using cold-temperature chemical treatments to maintain the structural collagen phase. However, the impact of collagen and the crystalline phase on physicochemical properties have not been elucidated. We hypothesized that understanding these factors could explain why the latter provides improved bone regeneration clinically. In this study, we compared two types of xenografts, one prepared through a low-temperature chemical process (Treated) and another subsequently sintered at 1100°C (Sintered) using advanced microscopy, spectroscopy, X-ray analysis and compressive testing. Our investigation showed that the Treated bone graft was free of residual blood, lipids or cell debris, mitigating the risk of pathogen transmission. Meanwhile, the sintering process removed collagen and the carbonate phase of the Sintered graft, leaving only calcium phosphate and increased mineral crystallinity. Microcomputed tomography revealed that the Treated graft exhibited an increased high porosity (81%) and pore size compared to untreated bone, whereas the Sintered graft exhibited shrinkage, which reduced the porosity (72%), pore size and strut size. Additionally, scanning electron microscopy displayed crack formation around the pores of the Sintered graft. The Treated graft displayed median mechanical properties comparable to native cancellous bone and clinically available solutions, with an apparent modulus of 166 MPa, yield stress of 5.5 MPa and yield strain of 4.9%. In contrast, the Sintered graft exhibited a lower median apparent modulus of 57 MPa. It failed in a brittle manner at a median stress of 1.7 MPa and strain level of 2.9%, demonstrating the structural importance of the collagen phase. This indicates why bone grafts prepared through cold-temperature processes are clinically favourable.

## Graphical abstract

**Figure:**
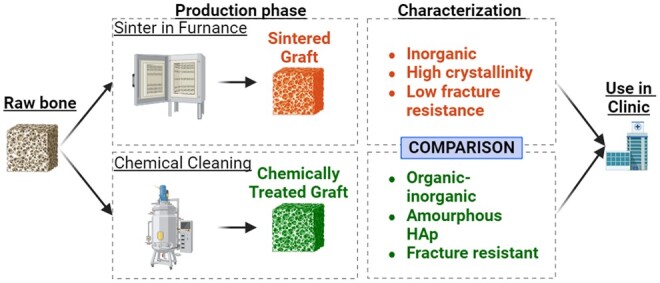

## Introduction

Bone regeneration needs are diverse, including dental, orthopaedic and spinal applications. A commonality, however, is that bone grafts quickly need to integrate with the native bone, not provoke any chronic inflammatory response or transfer of pathogenesis, and it needs to provide adequate mechanical support immediately [1]. In the case of dental application, e.g. sinus lift, grafts are typically used such that a dental implant can be inserted 6 months after the grafting procedure [2, 3]. For orthopaedic and spinal application, the graft must, in combination with any support plates, screws or cages, provide enough mechanical support to prevent movement that can inhibit fracture healing, e.g. for tibial plateau fractures [4]. In all cases, biological performance is crucial as quicker bone growth will increase patient satisfaction and can reduce healthcare costs. An ideal bone graft should exhibit osteoconductive properties, have a high porosity, and be able to take loading [5]. However, as pointed out by de Lacerda Schickert *et al*. [6], the biological and mechanical requirements for loading applications are often contradictory. Thus, a trade-off between these properties is required during the manufacturing process.

For animal-derived tissues, the European Medical Device Regulations (MDR—2017/745) and regulation 2012/722 on the use of medical devices of animal origin requires the tissue to be sourced in a manner that minimizes the risk of pathogens transmission by implementing validated methods for the inactivation or elimination of these pathogens during the manufacturing [7, 8]. The ISO 22442-series provides a reference for adequate risk management for these tissues. In terms of bovine-sourced grafts, the main concern is *bovine spongiform encephalopathy*, which the European Pharmacopoeia suggests can be inactivated or eliminated using sodium hydroxide, sodium hypochlorite or temperatures above 133°C [9]. Many producers sinter their grafts at temperatures typically ranging between 300°C and 1200°C, which does indeed remove any pathogens. Still, it also makes a xenograft fully inorganic by removing the collagen and changing the mineral structure to a high degree of crystallinity. This is the case with Bio-Oss^®^, the market-leading bone graft for dental applications in Europe, which uses temperatures up to 300°C during the sintering [10]. Newer methods produced deproteinized xenografts using process temperatures as low as 160°C, which can provide some advantages to Bio-Oss^®^ regarding improved bioresorption [11]. Native bone consists of low crystalline apatite and collagen, where the mineral crystals are nucleated and grow out of the collagen, yielding a composite material hierarchically organized down to a nanoscale [12]. This provides the combined high mechanical stiffness and high fracture toughness, where the collagen fibres are essential for the latter property [13]. The fracture toughness prevents catastrophic failure of the graft when exposed to loading, which a bone graft will typically experience after implantation.

Although many methods comprise high-temperature sintering [14], there are also possibilities for using biologically viable temperatures, maintaining the collagen and, thereby, the fracture toughness. There are also indications that they provide improved bone formation clinically [15]. Considering these cold-temperature methods for preparing allografts (human-derived) and xenografts, most cleaning procedures start with treatments to remove the bone marrow components from the structure. Over the last decade, a series of articles have started cleaning with 1% Triton X-100 (polyethylene glycol tert-octylphenyl ether), a non-ionic surfactant [16–21]. However, Triton X-100 has been put on the SVHC (Substances of Very High Concern) list according to the REACH (Registration, Evaluation, Authorisation and Restriction of Chemicals) regulation by the European Chemical Agency due to its endocrine-disrupting properties [22] and is therefore not suitable for preparation of bone grafts. The application of 3% hydrogen peroxide and 70% ethanol for additional fat removal has also been documented [17].

Other commonly used agents include sodium hydroxide for viral and prion inactivation and oxidizing agents such as hydrogen peroxide to eliminate cells and debris. However, both are recognized to have detrimental effects on the mineral and collagen structure of bone [23]. Therefore, the concentration and processing time should be kept to a minimum.

Tutoplast xenograft by Tutogen Gmbh utilizes a series of steps, including hydrogen peroxide, sodium hydroxide and acetone [23]. The process has been described in detail by Schoepf [24]: (i) removal of lipids with an ultrasonic acid bath, which is also claimed to reduce any prion load by two-log and inactivating viruses; (ii) rinse in baths of hyperosmotic salt water, which erupts the cell membrane, exposing any intracellular viruses and wash out cell debris, including bacteria; (iii) hydrogen peroxide is then used to eliminate soluble proteins, viruses and bacterial spores through oxidation; and (iv) a final acetone bath is used to reassure removal of prions and virus inactivation. After that, the graft is dehydrated using vacuum extraction before it is sterilized using low-dose gamma irradiation (17.8–20.1 kGy). Typically, for dental applications, allografts are consumed in the American market and bovine xenografts in the European market [5]. Bansal *et al*. [25] showed that xenograft blocks made with the Tutoplast method could support fracture union in tibia plateau fracture while maintaining mechanical integrity. Indeed, an elder patient group (average age of 74, *n* = 19) observed an average union time of 20 weeks and an average collapse of 4 mm. This demonstrates the relevance of non-sintered xenografts for orthopaedic application.

Currently, two xenografts are available in the clinic: sintered grafts and grafts are prepared through cold-temperature chemical cleaning. We hypothesize that the cold-temperature process is favourable as the collagen structure is maintained and the crystallinity of the apatite is not altered, but this has not been elucidated in the literature. This study compares bovine xenografts prepared by cold-temperature cleaning (Treated) or sintering (Sintered). The cleaning process of the treated graft has been optimized to remove bone marrow from any potential prions, kill bacteria and inactivate viruses while maintaining a polycrystal mineral structure and limiting damage to the collagen phase. We have compared the two grafts from a physicochemical, morphological and mechanical perspective, and we have applied cytotoxicity and endotoxin testing. By including clinically available controls, we have benchmarked our results and reassured the relevance of our work.

## Materials and methods

### Materials

All chemicals were ordered in pharmaceutical-grade versions from VWR Switzerland. Blocks of cancellous bovine bone from the femur condyle of young bulls were acquired from Rapelli SA (Stabio, Switzerland). Positive control groups were purchased directly from the supplier, used as supplied and were as follows: BTM^®^ granules of 0.25–1 mm (allograft—IOR Bologna, Italy), Tutogen blocks of 10 × 10 × 10 mm^3^ and granules of 0.25–1 mm (xenograft from Tutoplast^®^ process—Tutogen Medical GmbH, Erlangen, Germany) and Bio-Oss^®^ granules of 0.25–1 mm (deproteinized xenograft—Geistlich Pharma AG, Wolhousen, Switzerland). The Treated grafts were kindly prepared by Industrie Biomediche Insubri SA (Mezzovico-Vira, Switzerland) in ISO 13485:2016 compliant Good Laboratory Practice (GLP) facilities through a series of hypo/hyperosmotic soaks, solvent dehydration using polar aprotic solvent, lipid degradation with alkaline solution, such as sodium hydroxide solution, treatment with oxidating agent. The Treated grafts were produced in blocks of 10 × 10 × 10 mm^3^ or chips of 10 × 10 × 4 mm^3^. Negative control samples (‘untreated’) were obtained by centrifuging at 8000 rpm for 5 min to open porosity by extracting bone marrow. However, they did not undergo any chemical treatments.

### Preparation of samples for testing

The sintered graft (from now on Sintered) was prepared by sintering the Treated grafts for 1 h in a furnace at 1100°C at a heating and cooling rate of 5°C per minute (HTC-08/16, Nabertherm GmbH, Bremen, Germany). For cell testing, individual chips of Treated and Sintered grafts were packed in double pouching and sterilized using electron beam irradiation (25 kGry). For the recording of Fourier transformation infrared spectroscopy (FTIR), X-ray diffraction pattern (XRD) and transmission electron microscopy (TEM) analysis, the samples (except for Bio-Oss) were ground into a fine powder using a Mini-Mill PULVERISETTE 23 (FRITCH, Idar-Oberstein, Germany) ball mill.

### Attenuated total reflectance—Fourier transform infrared spectroscopy

ATR-FTIR spectra of the samples were collected on a Nicolet™ iS50 FTIR spectrometer (Varian Inc., Palo Alto, CA, USA) with the built-in ATR module. The spectra were collected in the mid-infrared range between 400 and 4000 cm^−1^ at a resolution of 4 cm^−1^ by co-adding 64 scans. Background spectrum with no sample in the infrared beam was acquired before the collection of the sample spectrum. Then, the background spectrum was subtracted from the sample spectrum. The absorption ratio at 1030/1110 cm^−1^ was used to calculate the crystallinity maturity ratio [26].

### Thermogravimetric analysis and differential scanning calorimetry

Simultaneous thermal analyser TGA/DSC 3+ (METTLER TOLEDO, USA) was used for TGA/DSC analysis of the samples. Approximately 45–60 mg of the sample was loaded into an alumina crucible and heated from 30°C to 1100°C at a rate of 10°C/min under flowing air (10 ml/min). The collagen content was determined by evaluating the mass change around the exothermic peak around 350–375°C, as previously established [27, 28].

### X-ray diffraction

The samples’ XRD patterns were recorded on a Malvern Panalytical Aeris (Malvern Panalytical, UK) XRD operated at 40 kV and 15 mA (Cu Kα radiation). Diffraction data were collected in a 10–70° 2θ range, with a step size of 0.04°. The total measurement time for each sample was 20 min. The material was identified by comparing peak position and intensity to a reference material of hexagonal hydroxyapatite [29, 30].

### X-ray microscopy

MicroCT was conducted with a Skyscan 1172 (Bruker-microCT, Kontich, Belgium) using a pixel resolution of 8.21 µm, and the X-ray source set at 64 kV and 148 µA, corresponding to a power of 9 W. A 360° scan was conducted with 0.53° steps, using an average of three frames per step. The reconstruction was done with Skyscan NRecon (Bruker-microCT, Kontich, Belgium). A ring artefact reduction of 8 was used for the reconstruction, along with a beam hardening correction of 60%. The signal was threshold between 0.02 and 0.75. Lastly, the post-processing was defined in CTan (Bruker-microCT, Kontich, Belgium) and executed in the batch processing software BatMan. For the post-processing definition, a volume of interest of 9743 px^3^ was chosen. From the processing, 3D morphological properties were derived along with the pore accessibility.

### Scanning and transmission electron microscopy

The surface morphology of the samples was investigated using scanning electron microscopy (SEM; Hitachi Analytical tabletop SEM TM3030, Hitachi, Japan). Samples were scanned at 15 kV and ×100 magnitude without using any coatings to be feasible to visually separate between mineral content and soft matter (fat and connective tissues, etc.). The SEM images were also coupled with energy-dispersive X-ray spectroscopy (EDS, Bruker, MA, USA; mag. ×500) to understand the local atomic composition. After the EDS analysis, the samples were sputter coated with Au for improved SEM image acquisition. Granulated samples were investigated using TEM (JEOL JEM-2100F, Tokyo, Japan) microscope with a Schottky field emission gun operated at 200 kV. The TEM images and diffraction patterns were acquired using a Hatan Orius 200D CCD (Gatan, Pleasanton, USA) camera, providing information about the nanoscale structure and crystallinity.

### Compressive testing

The apparent modulus (i.e. modulus of the porous structure treated as a homogenous material [31]) and yield point (using 0.2%-ε offset method) were characterized using a servo-hydraulic MTS testing machine (MTS 858 Systems Inc., Minneapolis, MN, USA) equipped with a 15 kN load cell. For the Sintered samples, changing the load cell calibration to a maximum of 1.5 kN was necessary. The cube samples (10 × 10 × 10 mm^3^) were placed between two flat plates, where the bottom one was attached to a ball joint to compensate for the cube surfaces potentially being unparallel. The samples were compressed at a rate of 1 mm/min till past the yielding point, as observed by the force level dropping or plateauing. It was conducted following number of repeats for each group: Untreated: 8; Treated: 8; Sintered: 8; Tutogen; 16; BTM: 7.

### Cytotoxicity and endotoxins

The cytotoxicity and endotoxins were measured using the exudates of the graft material, with the cytotoxicity testing conforming with ISO 10993-5 [32]. To prepare the exudates for cytotoxicity testing, the graft materials were incubated for 48 h at 37°C in cell medium (MEM with 15% serum and 1% antibiotic) at a concentration of 50 ml per gram of bone graft material. An osteoblastic cell line (MC3T3-E1) was seeded at a concentration of 2000 cells per well in a 96-well plate with 200 µl cell medium with eight repeats of each group. After being allowed to attach for 24 h in an incubator, the cell medium was removed and replaced with the exudate cell medium. After 48 h, the cell medium was extracted, and 100 µl of Cell Counting Kit 8 solution (mixed 1:45 with cell medium) was added to each well. After 1 h of incubation, the intensity was read off at 450 nm wavelength using a microplate reader (ELx800, BioTek, Vermont, USA). The viability was calculated according to Equation (1):

(1) $$\mathrm{Viability}\;\left(\%\right)=\frac{I_{s}}{I_{\mathrm{nc}}}\times 100,$$

where *I*_s_ was the mean sample intensity and *I*_nc_ was the mean control intensity. The 50 µl of each extract was added to a new 96-well plate and mixed with the 50 µl of a dye and catalyst mix from an Lactate dehydrogenase (LDH) cytotoxicity kit (Roche Diagnostics, Indianapolis, IN, USA). After 30 min of incubation at 37°C, the intensity was read off at 490 nm. The positive control was created by adding 1% of Triton X-100 1 h before the extraction. The cytotoxicity from the LDH assay was calculated according to Equation (2):

(2) $$\mathrm{Cytotoxicity}\;\left(\%\right)=\frac{I_{s}-I_{\mathrm{nc}}}{I_{\mathrm{pc}}-I_{\mathrm{nc}}}\times 100,$$

where *I*_pc_ is the intensity of the positive control, since exudate medium was used, the intensities were normalized by subtracting the mean intensity of the empty cell medium without any bonegraft from *I*_nc_ and *I*_pc_, and *I*_s_ was normalized by subtracting the mean intensity of the blank sample exudate. For endotoxin exudates of the grafts in endotoxin-free Phosphate-buffered saline (PBS) samples were prepared similarly as described above before the endotoxin levels were measured using a Pierce Chromogenic Endotoxin Quant kit (Thermo Fisher Scientific, Waltham, MA, USA) according to the instructions of the kit.

### Data processing and statistics

All data were analysed with custom Python scripts (Python 3.9), unless otherwise specified (e.g. µCT, SEM and TEM). The scripts depended on the libraries including Pandas, Numpy, Matplotlib, SciPy, sklearn, Seaborn and statannotations. Statistical differences were calculated using non-parametric Mann–Whitney *U* tests. A *P* values below 0.05 was considered statistically significant.

## Results

In this study, we investigated the physicochemical properties of xenographic bone grafts prepared either using a cold-temperature chemical treatment (referred to as ‘Treated’) and one prepared using subsequent sintering (referred to as ‘Sintered’). The objective was to gain a deeper understanding of how the preparation method for the xenografts affects its physiological properties, thereby obtaining an understanding of the role of the collagen and mineral crystallinity on the performance of the graft. We performed a comprehensive physicochemical characterization using techniques such as FTIR, XRD, TGA, TEM and compressive testing to achieve this. Furthermore, we tested cytotoxicity and viability on sterilized graft material using an LDH and CCK8 assay.

To make our research clinically relevant, we compared them against clinically available bone grafts, namely an allograft (BTM^®^), a xenograft (Tutogen), and a sintered, inorganic xenograft (Bio-Oss^®^). This comparison allowed us to assess our developed bone grafts’ suitability and potential advantages in relation to existing commercial options.

### Physicochemical characterization

**Figure 1.**
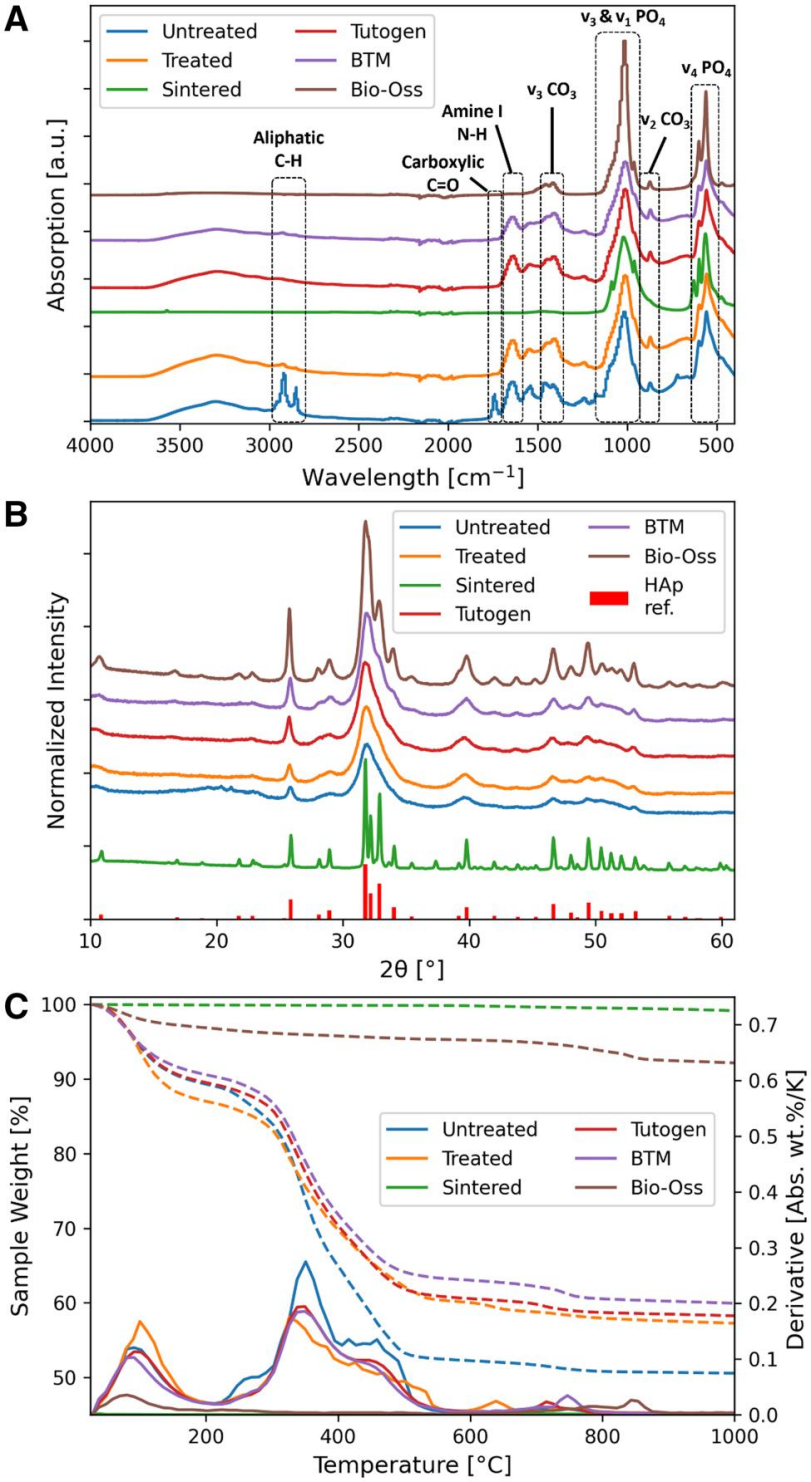
(**A**) ATR-FTIR plot of the treated and sintered grafts compared to untreated bone and benchmarked against three commercial bone grafts of allogenic (BTM^®^) and xenografic (Tutogen, Bio-Oss^®^) origin. (**B**) XRD patterns for the samples with a reference being hexagonal hydroxyapatite. (**C**) TGA plot for the samples, dotted lines representing the wt.% and the full line representing the derivative concerning temperature. HAp: hydroxyapatite.

ATR-FTIR was used to identify characteristic absorptions related to specific chemical bonds (Figure 1). At around 550 and 1000 cm^−1^, we observed the absorption bands of the v_3_ and v_1_  ${\text{PO}}_{4}^{3-}$, and at 1420–1490 and 870 cm^−1^ are the bands characteristic of carbonate vibrations. The phosphate bands were observable for all the grafts; however, the carbonate absorption bands were not for the Sintered graft. At 1550–1650 cm^−1^, the band associated with N-H bending vibration of primary amines can be observed. This is not observed for Bio-Oss^®^ nor the Sintered graft. The bands at 2900 and 2950 cm^−1^ are reported to be the aliphatic C-H stretches of residual fat, and the band at 1744 cm^−1^ is the v(C=O) stretching of the carboxyl in fatty acids molecules [14]. The fat-related absorption bands are evident in the untreated control sample (removal of bone marrow by centrifugation, but no chemical treatment) but not in the other samples. The mineral maturity ratio (FTIR 1030/1110 cm^−1^) is reported in Table 1, and it is significantly higher for the sintered samples (Sintered, Bio-Oss).

**Table 1.** Mineral maturity from the FTIR the phosphate band ratio 1030/1110 cm^−1^

Sample	**Mineral maturity (1030/1110** **cm**^−^**^1^)**
Untreated	2.10
Treated	2.65
Sintered	5.92
Tutogen	2.74
BTM	2.64
Bio-Oss	6.46

**Figure 2.**
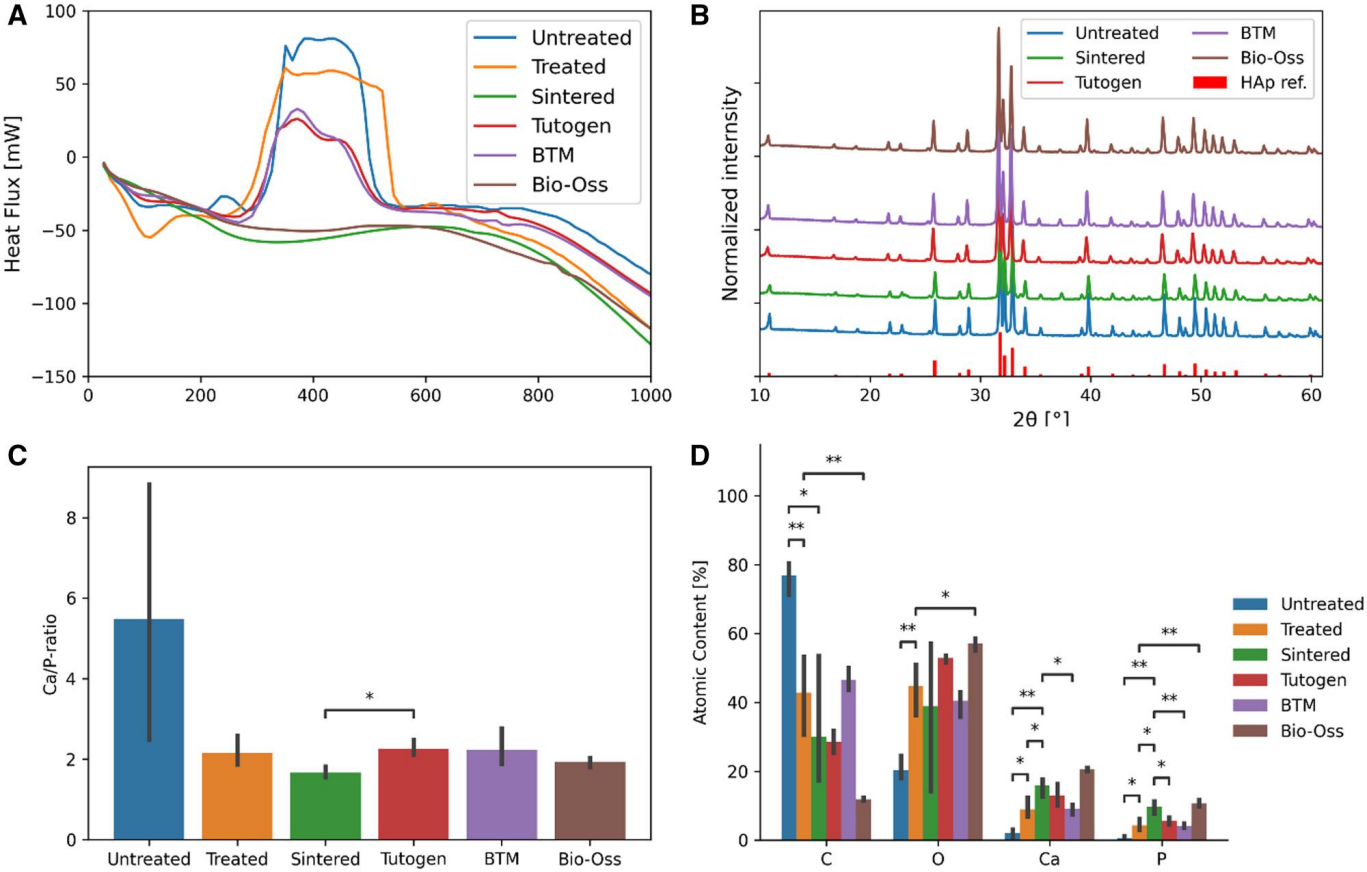
(**A**) DSC plot of the samples during the TGA analysis. The results suggest an endothermic reaction around 100°C, believed to be water evaporation, and an exothermic reaction starting between 350°C and 575°C, believed to be the combustion of collagen. (**B**) Of sintering of the bone grafts at 1100°C for 1 h. After sintering, the Treated graft is displayed as the Sintered graft. EDS results from the different grafts: (**C**) calcium–phosphorous ratio, (**D**) atomic % content of carbon, oxygen, calcium and phosphate. *P < 0.05, **P < 0.01, *n* = 3, mean ± 95% confidence interval for the EDS analysis.

All the non-sintered samples had similar XRD patterns with clear peaks matching that of hydroxyapatite reference peaks (Figure 1B), and tall narrow peaks for the sintered groups suggest higher crystallinity [33]. Also, the peaks of the sintered samples (Sintered, Bio-Oss) matched the reference peaks, however having narrower peaks with higher intensity and peaks, suggesting that the sintering increases the crystallinity of the sample. Interestingly, the Sintered sample had narrower peaks than Bio-Oss. We demonstrated that it was possible to increase the crystallinity of Bio-Oss further (Figure 2B). The Sintered graft also had a peak at 37.3 degrees (Figure 1B) when sintered also the BTM and Bio-Oss^®^ displayed a similar peak (Figure 2B), which can be caused by the calcination of calcium carbonate to calcium oxide [34]. From the TGA analysis (Figure 1C), we observed a significant mass decrease of ∼40 wt.% for the treated bone and 50 wt.% for the untreated bone (both after mechanical debridement of bone marrow). The first derivative reveals a peak at exactly 100°C for the treated bone, indicating water removal. The water content is ∼10 wt.% for the untreated bone and 12 wt.% for the treated bone, suggesting the presence of collagen that retains water. The untreated bone exhibits a broader peak, likely due to slower heat transfer caused by an insulating lipid layer. A second sharp peak appears at ∼375°C, which is believed to be due to collagen decomposition [35]. In the DSC results (Figure 2A), an exothermic process with heat release is observed between 350°C and 550°C, suggesting that this peak corresponds to the combustion of collagen. The mass drop around this peak indicates that the treated sample contains 27% collagen, while the untreated sample contains 35%. Additionally, there are two shoulders on the peak for the untreated bone, which can be attributed to the lipids from residual fat observed with FTIR.

Tutogen and BTM samples follow a similar curve to the treated sample but show a lower water peak, likely due to vacuum-drying and freeze-drying processes they underwent respectively, before they had a significant mass drop due to collagen combustion (Tutogen had roughly 30% collagen, BTM had 27%). In contrast, the treated sample has not undergone these treatments, although a vacuum-drying step is expected to be added during the technology transfer phase. Bio-Oss exhibits only a small water peak, likely due to ambient humidity, and no peak around 375°C. This supports the FTIR observation that Bio-Oss is inorganic, with collagen removed during the sintering process. The Sintered displayed negligible mass loss, suggesting it has been completely deproteinized and has lost the ability to store water. The material composition was also investigated with EDS (Figure 2C and D), which confirmed the presence of oxygen, carbon, calcium and phosphate in all groups, with negligible presence of other atoms. The carbon content is significantly higher for the untreated sample. In comparison, the calcium and phosphate content is significantly lower, likely due to the presence of a lipid layer from the sample surface where the reading is done. The Treated graft had a Ca/P-ratio of 1.93 ± 0.06, which dropped to 1.66 ± 0.13 after sintering. The others had a mean ratio from 1.93 (Bio-Oss) to 2.23 (BTM, Tutogen).

### Morphological characterization

The morphological analysis consisted of µCT, SEM and TEM, with the TEM also providing information about the crystallinity of the grafts. From the µCT it became evident that the treatment process increases the porosity of the bone, decreases the strut size and partially increases the pore size. Meanwhile, the subsequent sintering seems to increase the compactness of the graft again by reducing the pore size and the porosity (Table 2). When compared to the clinically available solutions, we can observe that the Treated sample exhibits a higher porosity than Tutogen, smaller struts and larger pores. Compared to BTM, the porosity of the Treated is similar, but the strut and pore size are smaller, suggesting that Treated has more but smaller but more pores than BTM. The sintered graft has a porosity similar to Tutogen’s, but the strut and pore sizes are significantly lower.

**Table 2.** µCT results of the bone samples before (Untreated) and after treatment (Treated) or sintering (Sintered)^a^

Parameter	Untreated	Treated	Sintered	Tutogen	BTM
Total porosity (%)	70.2 (68.5–80.8)	80.5 (80.2–81.9)	71.6*** (70.0–73.7)	74.3*** (71.4–76.2)	84.3^###^ (80.0–86.3)
Object surface/volume (mm^−1^)	18.8*(17.6–21.1)	23.1 (20.8–25.6)	22.6 (21.6–23.9)	18.0***^,^^##^^#^ (17.1–18.8)	21.9 (19.3–22.1)
Surface density (mm^−1^)	5.6^#^ (4.6–6.0)	4.3 (3.9–4.7)	6.4*** (5.9–6.7)	4.5^###^ (4.1–5.5)	3.4^###^ (3.1–4.3)
Strut thickness (µm)	168^#^ (154–177)	142 (130–161)	136 (130–139)	182***^,^^##^^#^ (175–192)	156 (151–169)
Pore size (µm)	473 (422–567)	600 (573–617)	409*** (386–450)	593^###^ (478–663)	718^###^ (610–775)

a Benchmarked against a xenograft (Tutogen) and an allograft (BTM). All samples were cubes of 1 cm^3^, from inside a volume of interest of 8 × 8 × 8 mm^3^ (6 × 6 × 6 for Sintered due to shrinkage) was used for the morphological analysis. Bio-Oss^®^ was not used in this study as it is only available as a fine powder. Median (IQR), *n* = 8 (*n* = 7 for BTM; *n* = 16 for Tutogen).

* *P* values < 0.05,

** *P* < 0.01,

*** *P* < 0.001 compared to Treated,

# *P* values < 0.05,

## *P* < 0.01,

### *P* < 0.001 compared to Sintered.

**Figure 3.**
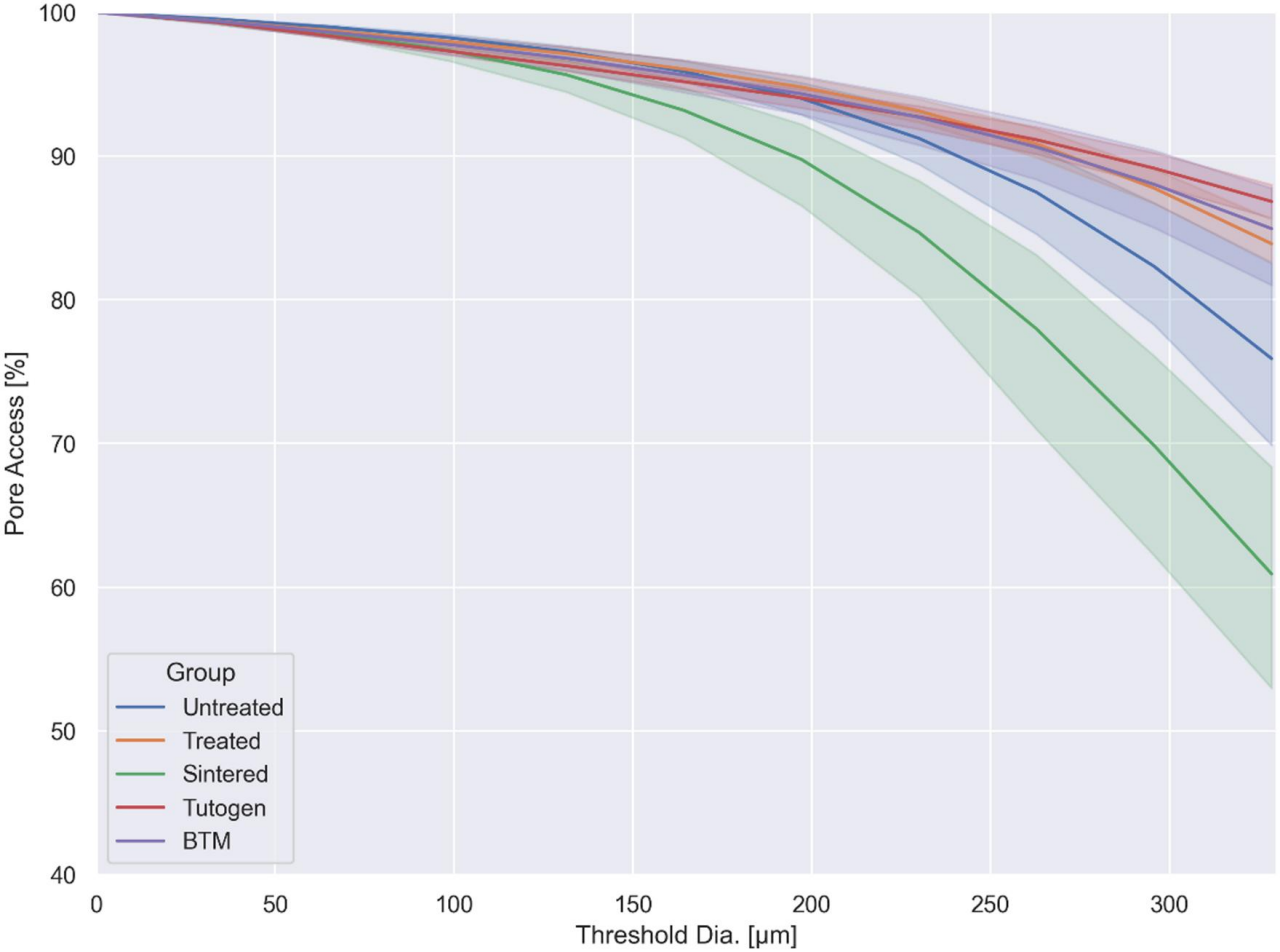
Interconnectivity plots with 95% confidence intervals.

The pore accessibility, commonly referred to as interconnectivity, was also investigated for different threshold diameters (Figure 3). For all groups, with the exception of the Sintered group, there was more than 90% pore access at a threshold of diameter of 200 µm. It can be observed that the Treated group had a slightly higher interconnectivity than the Untreated control. At the same time, it was drastically reduced for the Sintered group, suggesting that the cold-temperature cleaning process opens pores while Sintering shrinks the samples.

**Figure 4.**
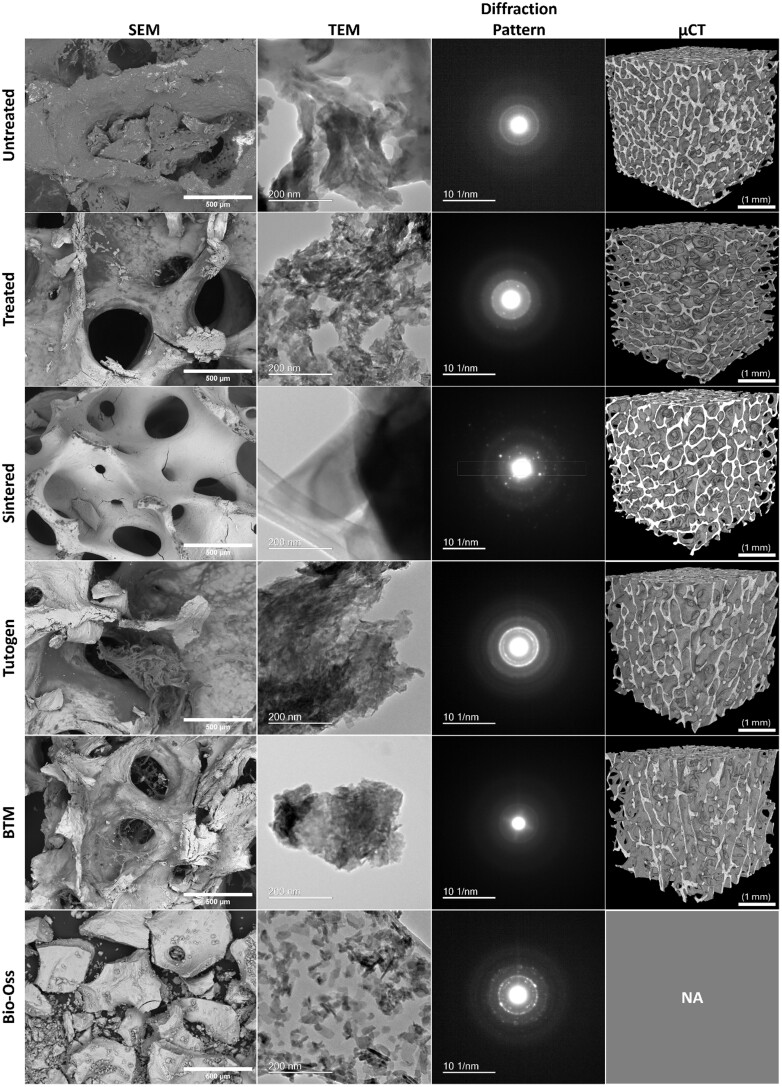
SEM, TEM images and diffraction patterns, and µCT reconstruction of untreated bone, Treated, Sintered, Tutogen, BTM and Bio-Oss^®^. Scalebars: SEM 500 µm; TEM 200 nm; diffraction pattern 10 nm^−1^; µCT 1 mm. µCT of Bio-Oss is omitted as it is only supplied in granular form.

The surface of the samples was characterized by SEM (Figure 4). In the untreated samples, it was evident that a layer of fat had still covered the surface. This was not the case in the remaining samples (Treated, BTM and Tutogen). The surface of the Treated samples seems rougher and has some cracks. This can be because the Treated sample’s surface was obtained through saw cutting. Meanwhile, the BTM and Tutogen samples were granules most likely prepared using a granulation machine. In the BTM samples, some strands of organic material can still be observed, but this is not observable in the Treated or Tutogen samples. No organic material from bone marrow was observed in the Bio-Oss and the Sintered samples. It all seems to have been removed. For the sintered graft, cracks can be observed forming around the pores. Bio-Oss are already provided in a granulated form, so they are not observable.

TEM further investigated the samples’ morphology and crystallinity (Figure 4). The untreated sample gave limited information as the lipids most likely interfered with the signal. Considering the non-sintered grafts (Treated, Tutogen and BTM), we can observe needle-like crystallites with a few nanometres in length. For the sintered graft, Bio-Oss, the crystallites are larger in the scale of tens of nanometres. Further observations can be made when evaluating the diffraction pattern from the TEM. The Untreated and BTM samples have a limited diffraction pattern, which can be due to the inorganic material we can observe from the SEM. Treated, Tutogen and Bio-Oss have clear diffraction patterns, and polycrystallinity is indicated in all samples. The Bio-Oss sample has a spottier pattern than the other samples, which can be related to the crystallinity or the crystallites’ size. The Sintered sample has large, thick crystallites and a spot diffraction pattern typical of a highly crystalline sample, which agrees with the observations from the XRD. Meanwhile, the remaining samples have more halo-like patterns. The halo-like patterns indicate more amorphous structures where the electrons are shattered in more directions than in a highly crystalline sample.

### Compressive strength

**Figure 5.**
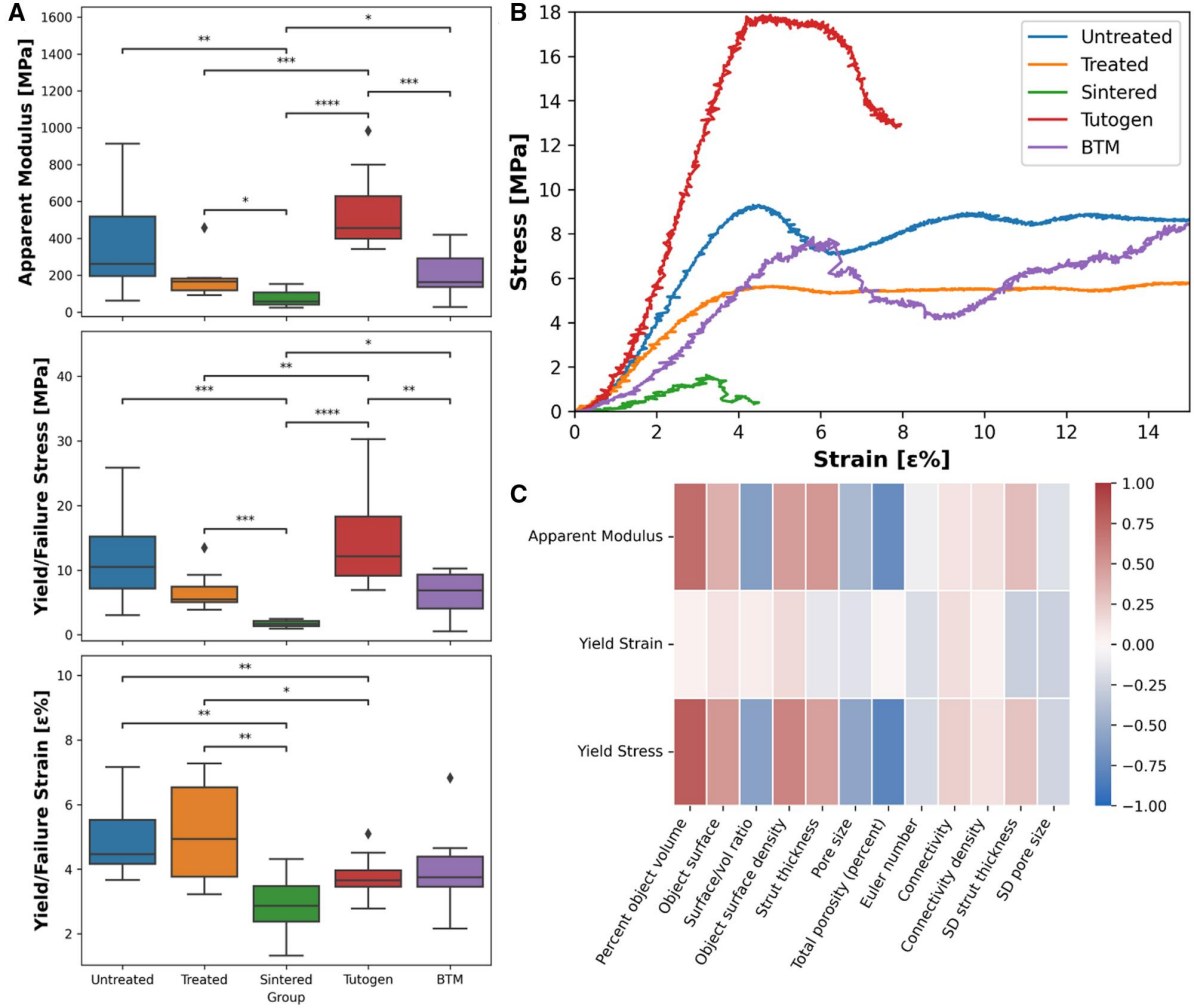
Results from uniaxial compressive testing coupled with µCT morphological data. (**A**) Mechanical parameters derived from uniaxial material testing (median and IQR—failure stress/strain for the sintered group). (**B**) Representative stress–strain plots for the different bone graft blocks. (**C**) Spearman ranking correlation test between mechanical properties (*y*-axis) and morphological properties (*x*-axis). Small correlation if 0.1 < |*r*| < 0.3; medium correlation if 0.3 < |*r*| < 0.5; and strong correlation if 0.5 < |*r*| < 1 [36] (the sintered group was not included in this analysis due to different failure mechanisms). *n* = [Untreated: 8; Treated: 8; Sintered: 8; Tutogen; 16; BTM: 7], *P < 0.05, **P < 0.01, ***P < 0.001, ****P < 0.0001.

The compressive testing was performed at a constant rate of 1 mm/min till past yielding (Figure 5A and B). All groups exhibited an initial linear region, before either yielding into a non-linear, ductile or in the case of the Sintered graft, failure in a brittle manner. This data derived the apparent modulus along with the yield stress and strain. In general, the data had a large variation due to the heterogenous structure of bone. It can be observed that after the cleaning process, both the apparent modulus (reduced from 261 to 166 MPa) and yield stress (reduced from 10.5 to 5.5 MPa) of the Treated graft have decreased compared to the untreated sample, but neither of the differences is statistically significant. For the sintered, the apparent modulus had been reduced to 57 MPa, and it had a failure stress of 1.7 MPa, which is statistically significant for both the treated group and untreated bone. Compared to the commercial controls, Treated’s apparent modulus and yield stress are significantly lower than Tutogen’s (461 and 12.1 MPa) but similar to BTM (162 and 6.9 MPa). However, before the treatment, the untreated bone had a lower modulus than Tutogen (261 vs 456 MPa), which can explain this. The Sintered graft had inferior apparent modulus and a lower failure stress than the yield stress of Tutogen and BTM, but a comparable failure strain (2.9 ε%) to their yield strain (3.7 and 3.7 ε%). The untreated and Treated bone had the best yield strain of 4.5 and 4.9 ε%, significantly higher than the other materials. To better understand the relationship between mechanical and morphological properties, the Spearman Rank Correlation Test was applied to the non-sintered groups (Figure 5C). The dominant relation seems to be a strong inverse relationship between the porosity and the apparent modulus (−0.74) and yield stress (−0.79), and a strong positive relation between solid volume and apparent modulus (0.72) and yield stress (0.81). Of course, the solid volume of the graft and porosity are directly linked to each other. Interestingly, the yield strain seems relatively independent from the morphological properties of the grafts.

### Cytotoxicity and endotoxins

**Figure 6.**
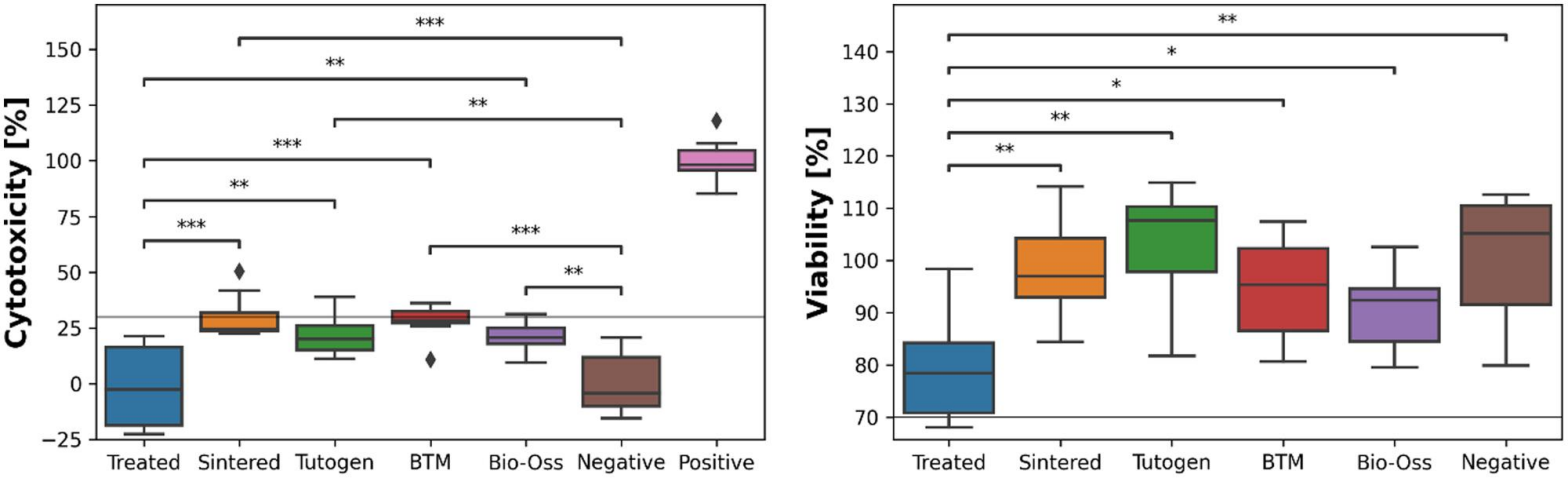
Cytotoxic effect of the graft exudates on MC3T3-E1 cell line conducted with a LDH (left) or CCK8 assay (right) after 48 h incubation. The black lines indicated the cytotoxic limit recommended by ISO 10993-5 of maximum 30% cytotoxicity or minimum 70% viability compared to the negative control, *p<0.05, **p<0.01, ***p<0.0001.

The cytotoxic effects of the exudates of the graft materials on an osteoblastic cell line (MC3T3-E1) were measured using two different assays (LDH and CCK8—Figure 6). In the case of the LDH assay, the Treated samples had a lower median cytotoxicity (−2.5%) than the other groups (Sintered: 24.6%, Tutogen: 20.0%, BTM: 28.5%, Bio-Oss: 20.6%) and comparable to the negative control group (−4.4%). The remaining groups had a higher median approaching the 30% limit of ISO 10993-5. The results were instead the opposite for the CCK8, which measures viability. The Treated group had significantly lower median viability (78.5%) than the other groups (Sintered: 97.0%, Tutogen: 107.6%, BTM: 95.4%, Bio-Oss: 92.4%) and the negative control (105.2%). The inter-quarter range of the treated group is still above the 70% viability limit. For endotoxins, all groups had a level lower than the detection limit of the kit.

## Discussion

In this work, we have compared two xenografts prepared either using cold-temperature chemical cleaning (Treated) or through sintering (Sintered), aiming to understand the differences in physicochemical properties and the role of collagen and crystallinity. To understand the clinical relevance of these treatment methods, we have compared these grafts to multiple clinically available solutions.

A central concern for the grafts independent of the preparation process is the effective removal of bone marrow, as the bone can be infected with pathogens such as viruses like HIV [37], bacteria [38] and prions [39]. Recently, there was a major outbreak of tuberculosis in the USA where 113 patients received an allografts containing live cells that were infected, causing the death of eight patients [40]. This is mainly a concern for cold-temperature treatment approaches, as high-temperature treatment is an efficient method to inactivate or remove these pathogens but can damage the collagen structure or make the bone inorganic. Chemical steps should be considered for the chemical treatment to inactivate any pathogen efficiently. There has been reports that sodium hydroxide is efficient at removing fat from bone materials [41], however from our visual inspections of SEM images of bovine xenografts treated with either 1 M sodium hydroxide for 15 min or 2 h in dichloromethane (Supplementary Figure S1), the organic solvent seems significantly more efficient at removing lipids from the graft. Sodium hydroxide is still essential for removing viral proteins and prions [41], but excessive exposure can compromise the graft. Based on their study, Dumas *et al*. [23] suggested that common anti-viral cleaning agents such as sodium hydroxide and hydrogen peroxide can induce deleterious changes to the mineral structure and the collagen, which *in vitro* retarded osteoblastic attachment, viability and other biological activities. This change to the bone structure would also likely reduce the mechanical properties of the graft. From our physicochemical analysis, in particularly from the FTIR, we demonstrated that the Treated graft was free for blood, lipids and other bone marrow residuals occupying the trabecular structure of the bone. Fortunately, the mineral structure of the Treated group is not evidentially damaged or modified by the process, and the amine peaks from the collagen can be readily observed, suggesting that the final graft is a native-like organic–inorganic composite structure. For the Sintered graft, there was an observable change in the chemical structure of the bone, suggesting removal of the collagen and the carbonate structure, leaving only apatite.

For the non-sintered grafts, we observed a considerable mass decrease of around 375°C with heat release during TGA, which we attribute to the combustion of collagen proteins. There is also a smaller peak between 700°C and 800°C, which can be attributed to the decomposition of calcium carbonate into calcium oxide [42]. Bio-Oss exhibited a similar peak but at around 840°C. Meanwhile, the sintered graft showed an insignificant weight change. Moreover, XRD before and after sintering the Treated graft (yielding the Sintered graft) at 1100°C for 60 min suggested that sintering significantly increased the crystallinity and crystallite size of the hydroxyapatite (narrower and taller peaks—Figure 2B) [33]. This conforms with previous results, suggesting that amorphous calcium phosphates will transform into crystalline phases upon heating above 600–650°C [43].

In our study, we used one sintered control, Bio-Oss. Although it exhibited significantly higher crystallinity than the other grafts and no collagen, the sintering at 1100°C further increased the crystallinity. The FTIR results also suggested a higher mineral maturity ratio for the sintered groups, corresponding to a transformation of the non-apatitic domain into apatitic domains [26], which appears to be triggered by sintering.

Nevertheless, in the clinic, Bio-Oss has been readily observable in histology samples even 4 years after implantation [33], likely due to the higher crystallinity, which makes it challenging for the body to resorb. For non-sintered grafts, e.g. SmartBone^®^, which is a polymer-coated product derived from the Tutogen graft, the remodelling occurs quickly, with clinical histology suggesting the graft is mostly resorbed after 6 months and fully resorbed after nine months [44]. Simultaneously, the SmartBone^®^ graft successfully stimulated the formation of new bone and vascularized connective tissue.

The morphological properties of the bone graft also have a significant impact on its performance. An ideal graft should have high porosity and interconnectivity to facilitate the diffusion of osteogenic cells, nutrition and removal of water products throughout the graft [5]. Our µCT results indicate that the chemical treatment of the Treated graft opens porosity and increases the interconnectivity of the graft, yielding a median porosity of over 80% and an interconnectivity of over 90% at a 200 µm cut-off threshold. This is significantly higher than for Tutogen and similar to BTM, both clinically successful grafts; thus, the results obtained can be interpreted as clinically favourable morphological properties.

However, an increased porosity will decrease the mechanical properties due to a reduction in solid volume, meaning that it tends to be a trade-off between morphological and mechanical properties. The mechanical properties of bone grafts are of great importance as most areas require bone regeneration to have intrinsic loading, both during static situations such as standing and particularly during dynamic events such as moving (for orthopaedics) and chewing (dental). It would be catastrophic if a graft failed, as this would require invasive revision surgery. Many bone grafts are inorganic ceramic structures [45, 46] or sintered xenografts [47], where the sintering removes the organic component of the bone. A critical limitation of ceramic bone grafts is that they tend to fail in a brittle manner, making them unsuitable for load-bearing applications [48]. There are indeed methods to mitigate this risk. In orthopaedics, a surgeon would typically implant metallic fixation plates [4, 49], which can off-load the graft from the load. However, this introduces a second foreign material implanted; it can cause stress-shielding, leading to asymmetric callus formation and fracture non-union [50], and it increases the risk of infection through biofilm formation on the metal surface [51]. Ideally, you would transfer as much loading as possible to the bone graft and limit the use of extra support devices, as the loading-induced mechanotransduction inhibits bone resorption and favours bone formation [52]. Reznikov *et al*. [53] observed in the femur model in sheep that there was an inverse relation between bone graft stiffness and bone regeneration and that this process was strain-driven in the animals that responded well to the therapy. However, they stressed major differences in the clinical response to the therapy. Thus, the graft still needs long-term mechanical integrity to handle the local loading profile. Our study obtained comparable apparent elastic modulus and yield stress for the Treated to BTM and somewhat lower than for Tutogen. However, the yield strain was greater in our case. Considering that the apparent modulus and yield stress were also lower for the untreated bone compared to Tutogen, it can indicate that the starting material of Tutogen was better than the one we used, which can relate to the bovine age and race. Independently of this, the apparent modulus and yield stress are within the range of human cancellous bone (100–500 and 0.1–30 MPa) [6, 54]. The compressive strength has been proven to decrease significantly when the process temperature surpasses 100°C, and as the process temperature is increased to 160°C and 220°C, further collagen is removed, decreasing the compressive strength [55]. The Sintered graft had significantly lower yield stress, roughly one magnitude, and the failure mechanism differed. Instead of exhibiting a linear regime followed by a stress plateau as the pores collapse, which is typical for cellular solids [46], it failed catastrophically, with the cracks propagating through the structure. As confirmed by FTIR and TGA, there is still a significant amount of collagen left in the non-sintered grafts, which can explain their ductile resistance.

In cases where the collagen is denatured or removed through heat treatment, this will reduce the grafts’ fracture toughness [56]. This clinically translates to that if a graft strut fails due to local stress levels surpassing the maximum stress, the crack would likely propagate through the whole structure, which can explain the shift towards cold-temperature processed grafts such as the Treated graft. As confirmed by FTIR and TGA, there is still a significant collagen content in the Treated graft, translating to a high-yield strain. However, the apparent modulus was significantly lower than Tutogen. We theorize this is due to the starting material initially having lower mechanical stiffness than the Tutogen graft. Compared to the BTM allograft there was no significant difference in either of the variables. Considering that both these grafts have been clinically adopted, we consider the Treated graft suitable for clinical applications. A limitation, however, is that we have not sterilized our samples prior to mechanical testing. Still, we are likely to do that using beta-ray sterilization, which can denature the collagen and reduce the toughness of the bone [57].

We coupled our understanding of mechanical and morphological properties on an individual sample level and conducted a Spearman Rank correlation test to improve our understanding of mechanical and morphological properties. Our results suggest that total porosity is the property that affects the mechanical properties (Apparent modulus: −0.74; yield stress: −0.79).

Yield strain only displayed a weak correlation to the morphological properties. We believe this is because collagen content is the prime variable affecting yield strain, as discussed above in the context of fracture toughness.

To get an early predictor of the grafts’ biocompatibility, their cytotoxic effect was measured using two assays, LDH and CCK8, and endotoxin levels were determined. DH measures cell death by looking at the release of lactate dehydrogenase, which is released when the plasma membrane of the cells is damaged [58]. The CCK8 kit measures the number of viable cells; thus, lower viability can suggest that the material inhibits cell growth. In our trials, we used the exudates of the graft material; thus, we did not directly measure the grafts’ cytotoxicity and cell viability on the material, but it gave us an indication of any potential leachables that can hamper biocompatibility. Conversely, it can also reduce the concentration of growth factors in the cell medium, which can inhibit cell viability. For the LDH assay, referring to cell membrane disruption, the Sintered and the control grafts had significantly higher cytotoxicity than the Treated group. However, they were still below the recommended 30% limit. For the viability test, the Treated graft performed poorer than the other groups and approached the lower limit of 70%, which we theorize that can be due to absorption of cell medium growth factors. It has previously been demonstrated that CCK8 can yield significantly lower cell viability results than with the commonly used 2,5-diphenyl-2H-tetrazolium bromide (MTT) method [59, 60]. All grafts displayed results within the acceptable range of ISO 10993-5, which is promising with the caveat that they do not necessarily give a thorough description of the grafts’ biocompatibility. Biocompatibility is a multi-faceted characteristic that comprises the lack of cytotoxicity, foreign response, and other adverse responses and the material’s ability to induce the appropriate biochemical cues for targeted cells to obtain the intended therapeutic effect [61].

For the graft materials investigated in this work, further investigation is required, including *in vivo* testing or advanced *in vitro* testing such as organoids, to see if and how the material can induce adequate bone formation in the clinic. A comprehensive understanding of the physicochemical properties of the graft is essential for elucidating phenomena and effects observed in biological systems, which was the motivation for this article. However, the path to clinical translation is extensive to ensure patient safety, also known as biocompatibility. The key steps are in conventional order to do *in vitro* cytotoxicity testing, animal trials to demonstrate efficacy and safety, and clinical trials before applying for regulatory approval is feasible [62]. In the USA, however, the clinical trials can be omitted using the 510(k) path if it can be proven substantial equivalence to a predicate device [62], which is feasible for the Treated graft as it should be classified as a Class II device by the United States Food and Drug Administration (FDA). We have demonstrated that the Treated’s comparable performance to the other non-sintered graft is promising for clinical translation.

Moreover, the cytotoxicity and endotoxin testing did not display any safety concerns. To translate this graft, it is necessary to continue *in vivo* trials before initiating clinical trials. Extensive *in vitro* trials can also be recommended to fully elucidate the cellular mechanisms the graft initiates. The Sintered graft exhibits compromised mechanical properties and a high crystallinity. Although there are no toxicity concerns, we are hesitant about its clinical potential due to inadequate elasticity and remodelling ability.

## Conclusion

This study comprehensively characterized and compared two xenografts, one prepared through cold-temperature chemical treatment and the other through sintering, to elucidate why the former provides improved clinical bone regeneration. Our findings indicate that the cold-temperature process preserves the native bone structure, with minimal changes to the calcium phosphate structure and limited collagen degradation. In contrast, sintering renders the bone graft inorganic phase and alters its crystallinity by removing all other phases than calcium phosphate. The sintered graft demonstrated significantly inferior mechanical properties compared to the treated graft, failing in a brittle manner and making it unsuitable for loading application. On the other hand, the Treated graft exhibited mechanical properties comparable to clinically available solutions, with no concerns regarding cytotoxicity or endotoxins. However, rigorous *in vivo* and clinical trials are required to translate these promising materials to clinical practice to ensure satisfactory biological performance. Given the desirable properties demonstrated in this study, we believe that the Treated graft can potentially become a clinically relevant solution.

## Supplementary Material

rbae093_Supplementary_Data

## References

[1] De Lauretis A , Øvrebø Ø, Romandini M, Lyngstadaas SP, Rossi F, Haugen HJ. From basic science to clinical practice: a review of current periodontal/mucogingival regenerative biomaterials. Adv Sci 2024;11:2308848. 10.1002/advs.202308848 PMC11077667 38380549

[2] Wychowanski P , Woliński J, Morawiec T, Kownacki P, Starzynska A, Kosieradzki M, Fiedor P. Preliminary clinical data and the comparison of the safety and efficacy of autogenous bone grafts versus xenograft implantations in vertical bone deficiencies before dental implant installation. Transplant Proc 2020;52:2248–51. 32252999 10.1016/j.transproceed.2020.02.099

[3] Mandelli F , Traini T, Ghensi P. Customized-3D zirconia barriers for guided bone regeneration (GBR): clinical and histological findings from a proof-of-concept case series. J Dent 2021;114:103780. 34400253 10.1016/j.jdent.2021.103780

[4] Ferracini R , Bistolfi A, Garibaldi R, Furfaro V, Battista A, Perale G. Composite xenohybrid bovine bone-derived scaffold as bone substitute for the treatment of tibial Plateau fractures. Appl Sci 2019;9:2675.

[5] Haugen HJ , Lyngstadaas SP, Rossi F, Perale G. Bone grafts: which is the ideal biomaterial? J Clin Periodontol 2019;46(Suppl 21):92–102. 30623986 10.1111/jcpe.13058

[6] de Lacerda Schickert S , van den Beucken JJJP, Leeuwenburgh SCG, Jansen JA. Pre-clinical evaluation of biological bone substitute materials for application in highly loaded skeletal sites. Biomolecules 2020;10:883. 32526829 10.3390/biom10060883 PMC7356650

[7] Council of the European Union, Medical Device Regulation 2017/745. 2017. URL: https://eur-lex.europa.eu/legal-content/EN/TXT/?uri=CELEX%3A32017R0745

[8] European Commission, COMMISSION REGULATION (EU) No 722/2012. 2012. URL: https://eur-lex.europa.eu/legal-content/EN/TXT/?uri=celex%3A32012R0722

[9] European Directorate for the Quality of Medicines & HealthCare, 5.2.8 Minimizing the risk of transmitting animal spongiform encephalopath agents via human and veterinary medicinal products, European Pharmacopoeia. 2019. URL: https://www.ema.europa.eu/en/documents/scientific-guideline/minimising-risk-transmitting-animal-spongiform-encephalopathy-agents-human-and-veterinary-medicinal-products_en.pdf

[10] Wenz B , Oesch B, Horst M. Analysis of the risk of transmitting bovine spongiform encephalopathy through bone grafts derived from bovine bone. Biomaterials 2001;22:1599–606. 11374460 10.1016/s0142-9612(00)00312-4

[11] Abdelmoneim D , Coates DE, Schmidlin P, Botter S, Li KC, Porter GC, Seo B, Duncan WJ. In vivo healing of low temperature deproteinized bovine bone xenograft in a rabbit cranial model. J Biomed Mater Res A 2024;112:1436–50. 38466022 10.1002/jbm.a.37693

[12] Reznikov N , Bilton M, Lari L, Stevens MM, Kroger R. Fractal-like hierarchical organization of bone begins at the nanoscale. Science 2018;360:eaao2189. 29724924 10.1126/science.aao2189 PMC6037297

[13] Stevens MM. Biomaterials for bone tissue engineering. Materials Today 2008;11:18–25.

[14] Ratnayake JTB , Gould ML, Shavandi A, Mucalo M, Dias GJ. Development and characterization of a xenograft material from New Zealand sourced bovine cancellous bone. J Biomed Mater Res B Appl Biomater 2017;105:1054–62. 26968590 10.1002/jbm.b.33644

[15] Schmitt CM , Doering H, Schmidt T, Lutz R, Neukam FW, Schlegel KA. Histological results after maxillary sinus augmentation with Straumann^®^ BoneCeramic, bio‐oss^®^, puros^®^, and autologous bone. A randomized controlled clinical trial. Clin Oral Implants Res 2013;24:576–85. 22324456 10.1111/j.1600-0501.2012.02431.x

[16] Coraça-Huber DC , Hausdorfer J, Fille M, Nogler M. Effect of storage temperature on gentamicin release from antibiotic-coated bone chips. Cell Tissue Bank 2013;14:395–400. 22936498 10.1007/s10561-012-9339-8

[17] Coraça-Huber DC , Hausdorfer J, Fille M, Steidl M, Nogler M. Effect of two cleaning processes for bone allografts on gentamicin impregnation and in vitro antibiotic release. Cell Tissue Bank 2013;14:221–9. 22581168 10.1007/s10561-012-9314-4

[18] Putzer D , Ammann CG, Coraça-Huber D, Lechner R, Schmölz W, Nogler M. The influence of liquids on the mechanical properties of allografts in bone impaction grafting. Biopreserv Biobank 2017;15:410–6. 28686464 10.1089/bio.2017.0003

[19] Putzer D , Dobersberger M, Pizzini A, Coraça-Huber D, Ammann C, Nogler M. Platelet concentrate as an additive to bone allografts: a laboratory study using an uniaxial compression test. Cell Tissue Bank 2018;19:559–67. 29855739 10.1007/s10561-018-9704-3 PMC6280855

[20] Putzer D , Huber DC, Wurm A, Schmoelz W, Nogler M. The mechanical stability of allografts after a cleaning process: comparison of two preparation modes. J Arthroplasty 2014;29:1642–6. 24793889 10.1016/j.arth.2014.03.028

[21] Kamra P , Lamba AK, Faraz F, Tandon S. Effect of antibiotic impregnation time on the release of gentamicin from cryopreserved allograft bone chips: an in vitro study. Cell Tissue Bank 2019;20:267–73. 30989363 10.1007/s10561-019-09765-8

[22] European Chemical Agency, Substance Infocard—Polyethylene glycol p-(1,1,3,3-tetramethylbutyl)Phenyl Ether. URL: https://echa.europa.eu/substance-information/-/substanceinfo/100.123.919 (2004, date last accessed).

[23] Dumas A , Gaudin-Audrain C, Mabilleau G, Massin P, Hubert L, Baslé MF, Chappard D. The influence of processes for the purification of human bone allografts on the matrix surface and cytocompatibility. Biomaterials 2006;27:4204–11. 16618501 10.1016/j.biomaterials.2006.03.044

[24] Schoepf C. The Tutoplast^®^ process: a review of efficacy. Zimmer Dental 2008;17:40–50.

[25] Bansal M , Bhagat S, Shukla D. Bovine cancellous xenograft in the treatment of tibial Plateau fractures in elderly patients. Int Orthop 2009;33:779–84. 18365191 10.1007/s00264-008-0526-y PMC2903103

[26] Farlay D , Panczer G, Rey C, Delmas PD, Boivin G. Mineral maturity and crystallinity index are distinct characteristics of bone mineral. J Bone Miner Metab 2010;28:433–45. 20091325 10.1007/s00774-009-0146-7 PMC2958843

[27] Lozano LF , Peña-Rico MA, Heredia A, Ocotlán-Flores J, Gómez-Cortés A, Velázquez R, Belío IA, Bucio L. Thermal analysis study of human bone. J Mater Sci 2003;38:4777–82.

[28] Durga R , Jimenez N, Ramanathan S, Suraneni P, Pestle WJ. Use of thermogravimetric analysis to estimate collagen and hydroxyapatite contents in archaeological bone. J Archaeol Sci 2022;145:105644.

[29] Markovic M , Fowler BO, Tung MS. Preparation and comprehensive characterization of a calcium hydroxyapatite reference material. J Res Natl Inst Stand Technol 2004;109:553–68. 27366634 10.6028/jres.109.042 PMC4856200

[30] Øvrebø Ø , De Lauretis A, Ma Q, Lyngstadaas SP, Perale G, Nilsen O, Rossi F, Haugen HJ. Towards bone regeneration: understanding the nucleating ability of proline-rich peptides in biomineralisation. Biomater Adv 2024;159:213801. 38401402 10.1016/j.bioadv.2024.213801

[31] Ghouse S , Reznikov N, Boughton OR, Babu S, Geoffrey Ng KC, Blunn G, Cobb JP, Stevens MM, Jeffers JRT. The design and in vivo testing of a locally stiffness-matched porous scaffold. Appl Mater Today 2019;15:377–88. 31281871 10.1016/j.apmt.2019.02.017 PMC6609455

[32] International Organization for Standardization, ISO 10993-5:2009 Biological evaluation of medical devices—Part 5: Tests for in vitro cytotoxicity. 2009. URL: https://www.iso.org/standard/36406.html (2004, date last accessed).

[33] Venkateswarlu K , Bose AC, Rameshbabu N. X-ray peak broadening studies of nanocrystalline hydroxyapatite by Williamson–Hall analysis. Phys B Condens Matter 2010;405:4256–61.

[34] Tangboriboon N , Kunanuruksapong R, Sirivat A. Preparation and properties of calcium oxide from eggshells via calcination. Mater Sci-Pol 2012;30:313–22.

[35] Pourjavadi A , Kurdtabar M. Collagen-based highly porous hydrogel without any porogen: synthesis and characteristics. Eur Polym J 2007;43:877–89.

[36] Cohen J. Set correlation and contingency-tables. Appl Psychol Meas 1988;12:425–34.

[37] Alexaki A , Wigdahl B. HIV-1 infection of bone marrow hematopoietic progenitor cells and their role in trafficking and viral dissemination. PLoS Pathog 2008;4:e1000215. 19112504 10.1371/journal.ppat.1000215 PMC2603331

[38] Binte Atique F , Khalil R, Masudur M. The bacterial contamination of allogeneic bone and emergence of multidrug-resistant bacteria in tissue bank. Biomed Res Int 2014;2014:430581. 25133161 10.1155/2014/430581 PMC4119633

[39] Takakura Y , Yamaguchi N, Nakagaki T, Satoh K, Kira J-I, Nishida N. Bone marrow stroma cells are susceptible to prion infection. Biochem Biophys Res Commun 2008;377:957–61. 18976632 10.1016/j.bbrc.2008.10.099

[40] Schwartz NG , Hernandez-Romieu AC, Annambhotla P, Filardo TD, Althomsons SP, Free RJ, Li R, Wilson WW, Deutsch-Feldman M, Drees M, Hanlin E, White K, Lehman KA, Thacker TC, Brubaker SA, Clark B, Basavaraju SV, Benowitz I, Burton Glowicz J, Cowan LS, Starks AM, Bamrah Morris S, LoBue P, Stewart RJ, Wortham JM, Haddad MB; Bone Allograft Tuberculosis Investigators. Nationwide tuberculosis outbreak in the USA linked to a bone graft product: an outbreak report. Lancet Infect Dis 2022;22:1617–25. 35934016 10.1016/S1473-3099(22)00425-X PMC9605268

[41] Bi L , Li DC, Huang ZS, Yuan Z. Effects of sodium hydroxide, sodium hypochlorite, and gaseous hydrogen peroxide on the natural properties of cancellous bone. Artif Organs 2013;37:629–36. 23373516 10.1111/aor.12048

[42] Karunadasa KS , Manoratne C, Pitawala H, Rajapakse R. Thermal decomposition of calcium carbonate (calcite polymorph) as examined by in-situ high-temperature X-ray powder diffraction. J. Phys Chem Solids 2019;134:21–8.

[43] Vecstaudza J , Gasik M, Locs J. Amorphous calcium phosphate materials: formation, structure and thermal behaviour. J Eur Ceram Soc 2019;39:1642–9.

[44] D'Alessandro D , Perale G, Milazzo M, Moscato S, Stefanini C, Pertici G, Danti S. Bovine bone matrix/poly (L-lactic-co-ε-caprolactone)/gelatin hybrid scaffold (SmartBone^®^) for maxillary sinus augmentation: a histologic study on bone regeneration. Int J Pharm 2017;523:534–44. 27769886 10.1016/j.ijpharm.2016.10.036

[45] Haugen H , Will J, Kohler A, Hopfner U, Aigner J, Wintermantel E. Ceramic TiO2-foams: characterisation of a potential scaffold. J Eur Ceram Soc 2004;24:661–8.

[46] Jelusic D , Zirk ML, Fienitz T, Plancak D, Puhar I, Rothamel D. Monophasic ß‐TCP vs. biphasic HA/ß‐TCP in two‐stage sinus floor augmentation procedures—a prospective randomized clinical trial. Clin Oral Implants Res 2017;28:e175–83. 27683073 10.1111/clr.12983

[47] Xu AT , Qi WT, Lin MN, Zhu YH, He FM. The optimization of sintering treatment on bovine‐derived bone grafts for bone regeneration: in vitro and in vivo evaluation. J Biomed Mater Res B Appl Biomater 2020;108:272–81. 31013400 10.1002/jbm.b.34387

[48] Laurencin C , Khan Y, El-Amin SF. Bone graft substitutes. Expert Rev Med Devices 2006;3:49–57. 16359252 10.1586/17434440.3.1.49

[49] Ferracini R , Bistolfi A, Guidotti C, Artiaco S, Battista A, Battiston B, Perale G. Bone loss in distal radial fractures treated with A composite xenohybrid bone substitute: a two years follow-up retrospective study. Materials (Basel) 2020;13:4040. 32933036 10.3390/ma13184040 PMC7558122

[50] Beltran MJ , Collinge CA, Gardner MJ. Stress modulation of fracture fixation implants. J Am Acad Orthop Surg 2016;24:711–9. 27579811 10.5435/JAAOS-D-15-00175

[51] Goodman SB , Yao Z, Keeney M, Yang F. The future of biologic coatings for orthopaedic implants. Biomaterials 2013;34:3174–83. 23391496 10.1016/j.biomaterials.2013.01.074 PMC3582840

[52] Oftadeh R , Perez-Viloria M, Villa-Camacho JC, Vaziri A, Nazarian A. Biomechanics and mechanobiology of trabecular bone: a review. J Biomech Eng 2015;137:10802. 10.1115/1.4029176 PMC5101038 25412137

[53] Reznikov N , Boughton OR, Ghouse S, Weston AE, Collinson L, Blunn GW, Jeffers JRT, Cobb JP, Stevens MM. Individual response variations in scaffold-guided bone regeneration are determined by independent strain- and injury-induced mechanisms. Biomaterials 2019;194:183–94. 30611115 10.1016/j.biomaterials.2018.11.026 PMC6345626

[54] Munford MJ , Ng KG, Jeffers JR. Mapping the multi‐directional mechanical properties of bone in the proximal tibia. Adv Funct Mater 2020;30:2004323.

[55] Abdelmoneim D , Porter GC, Coates DE, Duncan WJ, Waddell JN, Hammer N, Li KC. The effect of low-processing temperature on the physicochemical and mechanical properties of bovine hydroxyapatite bone substitutes. Materials 2022;15:2798. 35454491 10.3390/ma15082798 PMC9025514

[56] Burton B , Gaspar A, Josey D, Tupy J, Grynpas MD, Willett TL. Bone embrittlement and collagen modifications due to high-dose gamma-irradiation sterilization. Bone 2014;61:71–81. 24440514 10.1016/j.bone.2014.01.006

[57] Kaminski A , Grazka E, Jastrzebska A, Marowska J, Gut G, Wojciechowski A, Uhrynowska-Tyszkiewicz I. Effect of accelerated electron beam on mechanical properties of human cortical bone: influence of different processing methods. Cell Tissue Bank 2012;13:375–86. 22585354 10.1007/s10561-012-9312-6 PMC3432216

[58] Kumar P , Nagarajan A, Uchil PD. Analysis of cell viability by the lactate dehydrogenase assay. Cold Spring Harb Protoc 2018;6:465–9. doi: 10.1101/pdb.prot095497. 29858337

[59] Jiao G , He X, Li X, Qiu J, Xu H, Zhang N, Liu S. Limitations of MTT and CCK-8 assay for evaluation of graphene cytotoxicity. RSC Adv 2015;5:53240–4.

[60] Podgórski R , Wojasiński M, Ciach T. Nanofibrous materials affect the reaction of cytotoxicity assays. Sci Rep 2022;12:9047. 35641539 10.1038/s41598-022-13002-w PMC9156782

[61] Rahmati M , Silva EA, Reseland JE, C AH, Haugen HJ. Biological responses to physicochemical properties of biomaterial surface. Chem Soc Rev 2020;49:5178–224. 32642749 10.1039/d0cs00103a

[62] Øvrebø Ø , Perale G, Wojciechowski JP, Echalier C, Jeffers JR, Stevens MM, Haugen HJ, Rossi F. Design and clinical application of injectable hydrogels for musculoskeletal therapy. Bioeng Transl Med 2022;7:e10295. 35600661 10.1002/btm2.10295 PMC9115710

