# Supplementary material for: The role of collagen and crystallinity in the physicochemical properties of naturally derived bone grafts
Source: Regen Biomater. 2024 Aug 14;11:rbae093. doi: 10.1093/rb/rbae093 (PMC11368411; doi:10.1093/rb/rbae093)
Supplement: rbae093_Supplementary_Data [file rbae093_supplementary_data.docx]

**The role of collagen and crystallinity in the physicochemical properties of naturally derived bone grafts – Supplementary Material**

Øystein Øvrebø^1,2,3^, Luca Orlando^4,5^, Kristaps Rubenis^6,7^, Luca Ciriello^1^, Qianli Ma^2^, Zoe Giorgi^1^, Stefano Tognoni^1^, Dagnija Loca^6,7^, Tomaso Villa^1^, Liebert P. Nogueira^2,8^, Filippo Rossi^1^, Håvard J. Haugen^2,3^, Giuseppe Perale^4,9,10^

*Regenerative Biomaterials*, Volume 11, 2024, rbae093, <https://doi.org/10.1093/rb/rbae093>


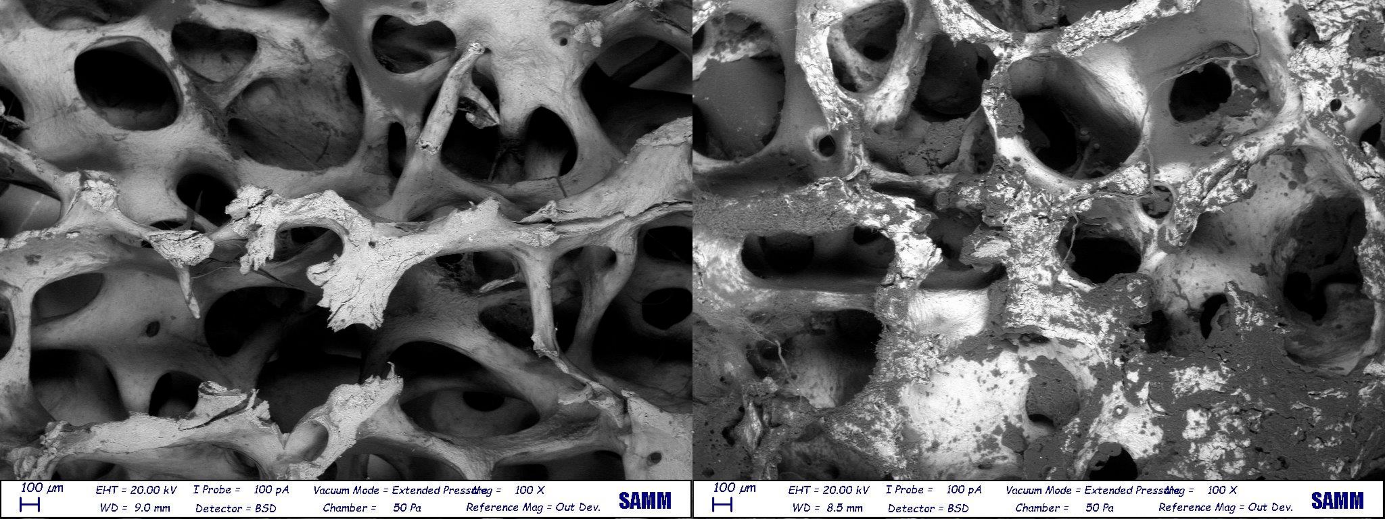


**Figure S1:** Xenografts treated with either DCM (left) or 1N sodium hydroxide (right). Bone marrow residuals can be observed as the dark phase on the sodium hydroxide sample, meantime the DCM sample seems completely bare from this.
